# Nanoscale Mechanical Properties of Nanoindented Ni_48.8_Mn_27.2_Ga_24_ Ferromagnetic Shape Memory Thin Film

**DOI:** 10.1155/2017/4630156

**Published:** 2017-05-28

**Authors:** Xiaofei Fu, Chao Liu, Xili Lu, Xianli Li, Jingwei Lv, Famei Wang, Liying Wang

**Affiliations:** ^1^The State Key Laboratory Base of Unconventional Oil and Gas Accumulation and Exploitation, College of Earth Science, Northeast Petroleum University, Daqing 163318, China; ^2^School of Electronics Science, Northeast Petroleum University, Daqing 163318, China; ^3^Institute of Materials Processing and Intelligent Manufacturing & Center for Biomedical Materials and Engineering, Harbin Engineering University, Harbin 150001, China

## Abstract

The structure and nanoscale mechanical properties of Ni_48.8_Mn_27.2_Ga_24_ thin film fabricated by DC magnetron sputtering are investigated systematically. The thin film has the austenite state at room temperature with the L2_1_ Hesuler structure. During nanoindentation, stress-induced martensitic transformation occurs on the nanoscale for the film annealed at 823 K for 1 hour and the shape recovery ratio is up to 85.3%. The associated mechanism is discussed.

## 1. Introduction

Ferromagnetic shape memory alloys have large magnetic-field-induced strain (MFIS) in addition to the magnetic shape memory effect (MSME) [1–3]. MFIS originates from the rearrangement of martensitic twin variants under an external magnetic-field or magnetic-field-induced martensitic transformation [4, 5] and up to 10% MFIS has hitherto been observed from Ni-Mn-Ga single crystals [6]. Researchers working on microelectromechanical systems (MEMS) have recently turned their attention to Ni-Mn-Ga ferromagnetic shape memory alloy (SMA) thin films due to potential applications microdevices and actuators [7–15]. Despite more understanding about the preparation process [8–10], phase transformation [11, 12] and magnetic transport properties [13–15] of Ni-Mn-Ga thin films, the nanoscale mechanical properties which are crucial to MEMS and involved in stress-induced martensitic transformation and shape recovery ratio on the Nanoscale have not been extensively explored. In this work, nanoindentation is conducted on Ni_48.8_Mn_27.2_Ga_24_ thin films and stress-induced martensitic transformation is observed. This study provides insights to the nanomechanical characteristics of Ni-Mn-Ga thin films which have large potential in MEMS.

## 2. Experimental Details

A Ni-Mn-Ga film 4 *μ*m thick was deposited onto a single crystal Si (100) substrate by DC magnetron sputtering at 300 W power and 0.4 Pa working pressure. The composition of the target was Ni_47_Mn_30_G_23_ with a diameter of 60 mm and thickness of 2 mm. Before deposition, the base pressure was better than 2 × 10^−4 ^Pa and the target was presputtered for at least 30 minutes to eliminate the surface oxide layer. Deposition was conducted for 90 minutes and the substrate was not heated. In order to crystallize the as-deposited Ni-Mn-Ga films, they were annealed at 723 K, 773 K, or 823 K for 1 hour at a reduced pressure of 4 × 10^−4 ^Pa.

The composition of the as-deposited Ni-Mn-Ga thin film was determined by energy-dispersive X-ray spectroscopy (EDS) equipped with the scanning electron microscope (SEM, S-4700). The crystallographic structure was analyzed by X-ray diffraction (XRD) using a piece of free-standing Ni-Mn-Ga thin film peeled off from the silicon substrate and annealed at 773 K for 1 hour at 3.0 × 10^-4 ^Pa. The size of the peeled thin film is approximately 7 mm × 7 mm. The martensitic transformation temperature is attempted to test by means of differential scanning calorimetry (DSC, Perkin-Elmer Diamond). The mass of the free-standing thin film is approximately 1 mg for DSC measurements. The microstructure was observed by transmission electron microscopy (TEM, JEOL 3000F, 300 kV). The thin foil was prepared by twin-jet electropolishing in an electrolyte containing nitric acid and methanol (3 : 7 in volume) at 250 K. The nanoscale mechanical properties were determined on a Hysitron triboscope nanoindenter in conjunction with a Digital Instruments nanoscope IV atomic force microscope (AFM). The nanoindenter was equipped with a Berkovich triangular pyramidal tip with the size of 200 nm and attached to the AFM scanner tube, thus allowing in situ topographical imaging at small loads. A suitable area was selected and the indentation experiments were performed within a few nanometers of this area using a maximum load of 9 mN.

## 3. Results and Discussion

**Figure 1:**
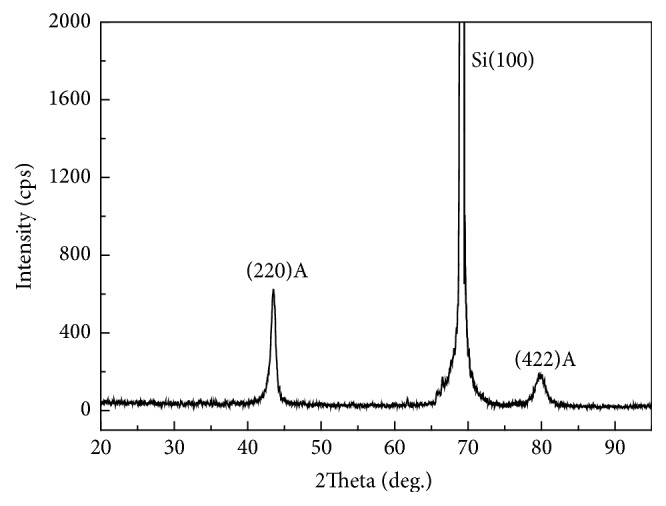
Typical XRD pattern of the Ni_48.8_Mn_27.2_Ga_24_ thin film annealed at 823 K for 1 hour.

**Figure 2:**
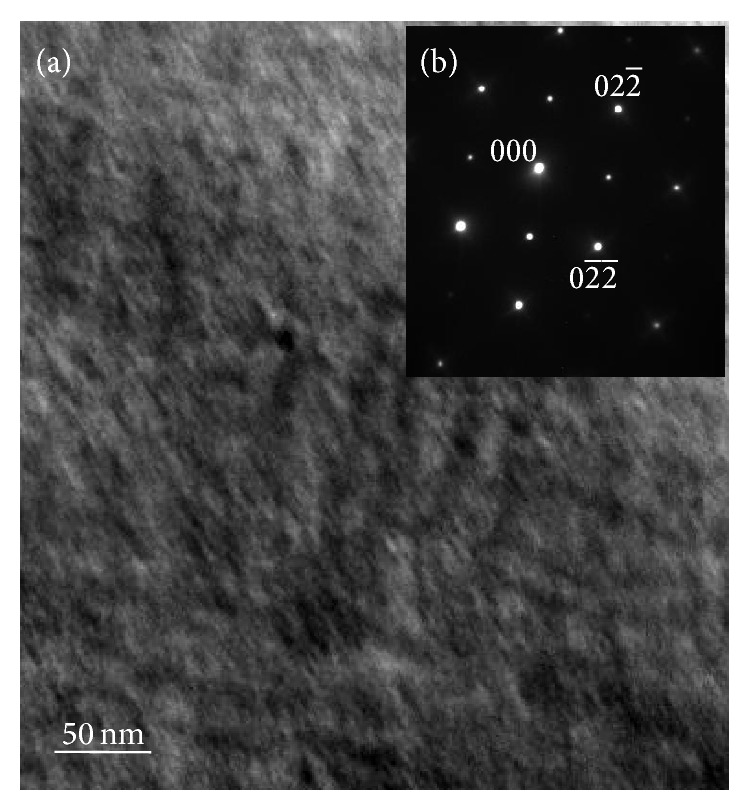
Microstructure of the parent phase in the thin film annealed at 823 K for 1 hour: (a) Bright field image and (b) EDP taken from (a) with the electron beam along [100].

The composition of the Ni-Mn-Ga thin film is Ni_48.8_Mn_27.2_Ga_24_. In order to determine the martensitic transformation temperature, DSC is attempted but no evident phase transformation peaks are detected due to a low thermal enthalpy and small mass (~1 mg) of thin film peeled off from the substrate [9]. To further determine the phases, the representative XRD pattern of the Ni_48.8_Mn_27.2_Ga_24_ thin film annealed at 823 K for 1 hour is depicted in Figure 1. Only one diffraction peak at approximately 43.9° can be observed and it can be indexed to the cubic austenitic structure. In addition, the film annealed at 723 K and 773 K for 1 hour is also cubic austenitic structure. The microstructure of the film annealed at 823 K for 1 hour is assessed by TEM (Figure 2). As shown in Figure 2(b), the electron diffraction pattern (EDP) suggests a superlattice structure and the indexed result further reveals that the thin film has the austenite state with the L2_1_ Hesuler structure.

**Figure 3:**
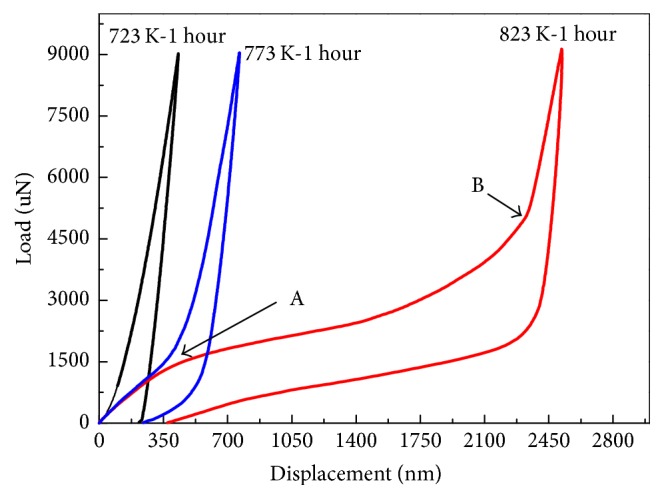
Nanoindentation curves of the thin film annealed at 723 K, 773 K, and 823 K for 1 hour under a maximum load of 9 mN at room temperature.

Figure 3 shows the load versus indentation depth curves of the specimens annealed at 723 K, 773 K, and 823 K for 1 hour under a maximum load of 9 mN at room temperature. The overall shape of the load-depth curves is different. With regard to the specimen annealed at 723 K, the load-depth curve indicates that the specimen is relatively hard and possesses relatively high resistance to the indenter. The nonlinear unloading path of the load-depth curve is consistent with measurements conducted on brittle materials [16, 17]. For the specimen annealed at 773 K, the initial portion of the load-depth curve increases gradually followed by a sharp increase. During unloading, nonlinear pseudoelasticity recovery is observed, indicating relatively soft and elastic characteristics which are not similar to those of the specimen annealed at 723 K. As the annealing temperature is raised to 823 K, a wide plateau can be observed from the load-depth curve suggesting stress-induced martensitic transformation on the nanoscale. The reason for the absence of stress-induced martensitic transformation in the specimens annealed at 723 K and 773 K may be attributed to the existence of the amorphous phase in the films annealed at low temperature. The amorphous phase in the films makes it difficult to accommodate the load and the transition between austenite and martensite. In addition, it is important to note that the indentation response of the thin films on silicon substrates is a complex function of the elastic and plastic properties of both the film and substrate. In particular, the indentation depth for the film annealed at 823 K is more than 50% of the film thickness. Silicon substrate effects are thought to play a major role in the overall load-displacement deformation processes that occur around the indenter. These possible effects include the elastic and plastic properties of silicon substrates, size effects in indentation plasticity, and dislocation interactions in small volumes [18–20]. These are clearly challenges for future work.

In stress-induced martensitic transformation, the load-depth curve can be divided into three stages upon loading. The initial portion of the curve at an indentation depth of below 200 nm is purely elastic deformation of the austenite phase, which is in accordance with the Hertz contact theory [21]. The elastic indentation force of the parent phase (*F*_aus_) is determined by the following equation [21]:

(1) $$\begin{array}{c} F_{aus}=\frac{4}{3}\left(\;{E_{a}}^{*}R^{1/2}h^{3/2}\;\right), \end{array}$$

where *E*_*a*_^*∗*^ = *E*_*a*_/(1 − *ν*^2^_*a*_), *R* is a constant related to the indenter tip,* h *is the depth, *E*_*a*_ is the elastic Young's modulus of austenite, and *ν*_*a*_ is the Poisson ratio of austenite. Larger indentation loads result in yield of austenite because austenite is unable to accommodate the additional deformation. That is, when the equivalent stress is involved in the indentation stress, the residual stress gradients originating from annealing reach the martensitic transformation stress and nucleation of the low symmetry martensite phase is impelled driving the lattice distortion of the parent phase into martensite. It can be seen that the yield point A (load of 1500 uN) in Figure 3 is the critical transition point of the austenite-to-martensite phase transformation. The transformation stress on the nanoscale has a magnitude of about 2 mN. After the yield point A, the slope of the load-displacement curve decreases remarkably and exhibits a wide plateau. The plateau, that is, the second stage, is ascribed to the reorientation of martensitic twin variants and formation of a single variant. The third stage of the curve upon loading arises from the elasticity of the single variant. During unloading, the specimen undergoes stress-induced reverse martensitic transformation as evidenced by the stress hysteresis plateau which corresponds to the elastic recovery of the single martensitic variant. Afterwards, the reverse martensitic transformation takes place with decreasing loads due to the elastic energy stored in the martensitic single variant as the driving force [22].

**Figure 4:**
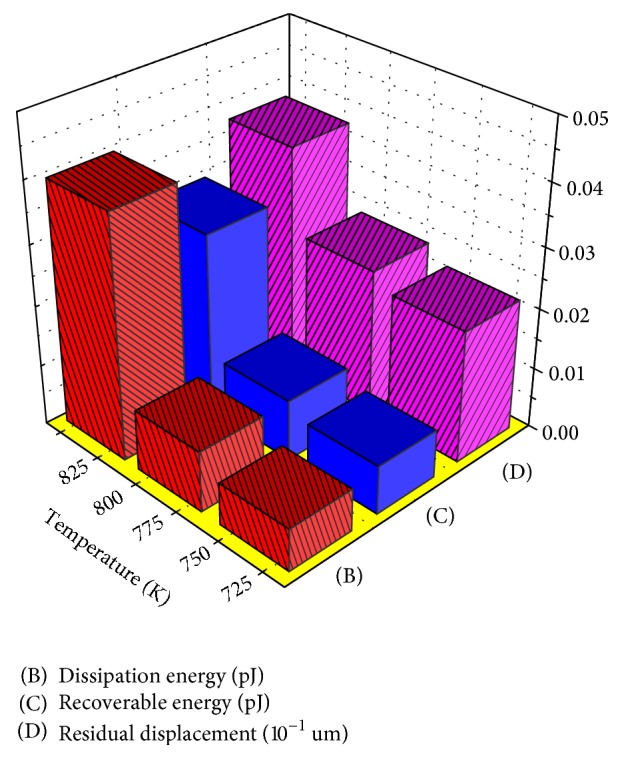
Dissipation energy and residual displacement of the thin film annealed at 723 K, 773 K, and 823 K for 1 hour after unloading.

The load-depth curves are characterized by the stress hysteresis. The area under the unloading portion of the indentation load-depth curve determines the recoverable energy corresponding to the lattice relaxation and reverse transformation [23]. The area between the loading and unloading portions of the indentation load-depth curve determines the dissipated energy [23]. Hence, there is stress hysteresis in the indentation curves. The dependence of the dissipation energy and recoverable energy as well as residual depth on the annealing temperature is illustrated in Figure 4. The dissipation energy and residual depth of the specimen annealed at 823 K are larger than those of the specimens annealed at 723 K and 773 K. The larger energy dissipated in the specimen annealed at 823 K is to overcome the residual compressive stress induced during annealing and stress-induced martensitic transformation. The stress-induced martensite crystals in the parent phase require energy to obtain energetically favorable orientations in nucleation and growth. Moreover, a portion of the dissipated energy contributes to strain mismatch at the martensite-austenite boundaries during martensitic transformation and reverse transformation. As a consequence, the energy dissipated for stress-induced martensitic transformation is larger than that of specimens without stress-induced martensitic transformation.

**Figure 5:**
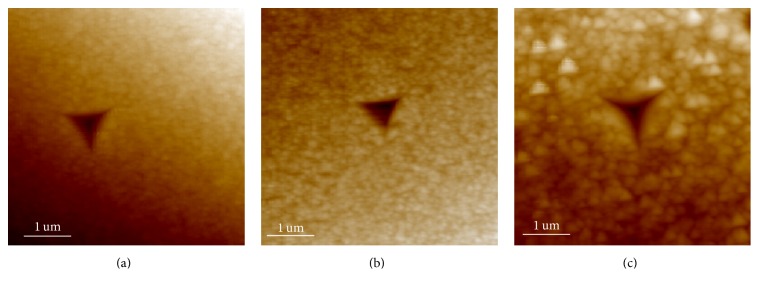
AFM indentation impressions in the annealed thin film after unloading: (a) 723 K for 1 hour; (b) 773 K for 1 hour; (c) 823 K for 1 hour.

Figure 5 presents the indentation impressions on the annealed specimens after unloading. The area and depth of the residual impressions as determined by in situ AFM increase with annealing temperature. In order to quantify the effects of elastic and pseudoelastic recovery, a recovery ratio *R* is expressed by the following formula:

(2) $$\begin{array}{c} R=\frac{\left(D_{max}-D_{res}\right)}{D_{max}}\times 100\%, \end{array}$$

where *D*_max_ is the maximum indentation depth and *D*_res_ is the depth of the residual indent.* R *signifies the combined effects of elastic and pseudoelastic recovery. The recovery ratios *R* of the specimens annealed at 723 K, 773 K, and 823 K are 49.2%, 68.5%, and 85.3%, respectively. For the specimens annealed at 723 K and 773 K, the lower recovery ratio of less than 70% reveals high resistance to the indenter, whereas the specimen annealed at 823 K displays good accommodating capability due to stress-induced martensitic transformation. Although the stress-induced martensitic transformation occurs in the specimen annealed at 823 K, there is residual deformation after unloading, mainly because of the irreversible strain between nucleated martensite and austenite as well as influence by the sharpness of the indenter tip [24]. Additionally, the surface roughness of the specimens annealed at 723 K, 773 K, and 823 K is 18.4 nm, 20.6 nm, and 42.6 nm, respectively. The surface roughness increases with the annealing temperatures increase. Larger surface roughness causes stress concentrations that are beneficial to trigger martensitic transformations in films [25]. Therefore, the thin film annealed at 823 K exhibits a stress-induced martensitic transformation in the load-displacement curve, as shown in Figure 3. It has been shown that it is more difficult for the remnant impressions from microscale sharp indenters to recover compared to those from spherical indenters due to the higher stress levels inherent to a sharp contact [24]. In addition, the depth of indentation is up to 2,500 nm in the specimen annealed at 823 K and it is about three orders of magnitude larger than that of the completed recovery in TiNi thin films [26]. Therefore, it can be concluded that despite the residual impression left after unloading for the specimen annealed at 823 K, this specimen possesses a good ductility because that the load can be accommodated by the elasticity of austenite and reorientation of martensitic twin variants induced by the load.

## 4. Conclusion

Nanoindentation is conducted to determine the compressive properties of Ni-Mn-Ga ferromagnetic shape memory alloy thin film on the nanoscale. Stress-induced martensitic transformation is observed from the Ni_48.8_Mn_27.2_Ga_24_ thin film at room temperature, and after annealing at 823 K for 1 hour, a relative large shape recovery ratio of 85.3% is achieved. The results suggest the potential use of Ni-Mn-Ga thin films in MEMS.
